# The impact of secondhand smoke on the development of kidney stone disease is not inferior to that of smoking: a longitudinal cohort study

**DOI:** 10.1186/s12889-023-16116-6

**Published:** 2023-06-20

**Authors:** Yi-Hsuan Chen, Jia-In Lee, Jung-Tsung Shen, Yi-Hsuan Wu, Yao-Hsuan Tsao, Jhen-Hao Jhan, Hsun-Shuan Wang, Yung-Chin Lee, Shu-Pin Huang, Szu-Chia Chen, Jiun-Hung Geng

**Affiliations:** 1 Department of Urology, Kaohsiung Medical University Hospital, Kaohsiung Medical University, Kaohsiung, Taiwan; 2 Department of Urology, Kaohsiung Municipal Ta-Tung Hospital, Kaohsiung, Taiwan; 3 Department of Urology, Kaohsiung Municipal Siaogang Hospital, Kaohsiung, Taiwan No. 482, Shanming Rd, Xiaogang District, 812; 4 Department of Psychiatry, Kaohsiung Medical University Hospital, Kaohsiung Medical University, Kaohsiung, Taiwan; 5 Graduate Institute of Clinical Medicine, College of Medicine, Kaohsiung Medical University, Kaohsiung, Taiwan; 6 Faculty of Medicine, College of Medicine, Kaohsiung Medical University, Kaohsiung, Taiwan; 7 Ph.D. Program in Environmental and Occupational Medicine, College of Medicine, Kaohsiung Medical University, Kaohsiung, Taiwan; 8 Department of Internal Medicine, Kaohsiung Municipal Siaogang Hospital, Kaohsiung Medical University, Kaohsiung, Taiwan; 9 Division of Nephrology, Department of Internal Medicine, Kaohsiung Medical University Hospital, Kaohsiung Medical University, Kaohsiung, Taiwan; 10 Research Center for Environmental Medicine, Kaohsiung Medical University, Kaohsiung, Taiwan

**Keywords:** Smoking, Secondhand smoke, Kidney stone disease, Urolithiasis, Risk factor, Epidemiology, Longitudinal cohort, Taiwan Biobank

## Abstract

**Background:**

Tobacco use and secondhand smoke (SHS) are risk factors of kidney stone disease (KSD). The hypothesis is that tobacco produces chemicals that increase oxidative stress and vasopressin, which leads to decreased urine output, and contributes to stone formation. The aim of this study was to examine the effects of smoking and SHS on the development of KSD.

**Materials and methods:**

We analyzed a total of 25,256 volunteers with no history of KSD participated in the Taiwan Biobank. The presence of underlying and follow-up KSD was surveyed by a self-administrated questionnaire. They were classified into three groups on the basis of smoking and SHS exposure, accessed with survey questionnaires; never-smokers with no SHS exposure, never-smokers with SHS exposure and ever-smokers groups.

**Results:**

KSD was noted in 352 (2.0%), 50 (3.3%) and 240 (4.1%) subjects in the never-smokers with no SHS exposure, never-smokers with SHS exposure and ever-smokers groups, respectively, with a mean follow-up of 4 years. The odds ratio (OR) of KSD was higher in the never-smokers with SHS exposure (OR, 1.622; 95% confidence interval [95% CI], 1.225 to 2.255) and ever-smokers groups (OR, 1.282; 95% CI, 1.044 to 1.574) than in the never-smokers with no SHS exposure group after adjustment of confounders. In addition, never-smokers with SHS exposure had similar effects on the development of KSD than ever-smokers (OR, 1.223; 95% CI, 0.852 to 1.756).

**Conclusion:**

Our study suggests that both smoking and SHS are a risk factor for developing KSD and that the impact of SHS is not inferior to that of smoking.

**Trial registration:**

The study was conducted in accordance with the Declaration of Helsinki and approved by the Institutional Review Board of Kaohsiung Medical University Hospital (KMUHIRB-E(I)-20,210,058).

**Supplementary Information:**

The online version contains supplementary material available at 10.1186/s12889-023-16116-6.

## Background

Kidney stone disease (KSD) is a common renal disease around the world and the prevalence rates vary from 5 to 9% in Europe, 7-13% in North America, and 1–5% in Asia [1]. In Taiwan, the incidence rate of KSD is up to 10% throughout lifetime and has increased in recent decades [2]. KSD can contribute to pain, infection, urinary tract obstruction, thus affecting patients’ kidney function, quality of life and comorbidities, which have a huge impact on human health [3]. There are many factors associated with the development of KSD, including age [4], male gender [5], obesity [6], genetics [7], environmental pollutants [8], habits and customs [9]. By avoiding the modifiable risks factors may promote human health and reduce medical burden.

Tobacco use is a major cause of death from pulmonary disease, cardiovascular disease and cancers, which becomes a worldwide public health major issue [10]. More importantly, smoking not only causes harm to the smoker itself, but also harms those who inhale the smoke from the smoke breathed out by smokers or from the burning end of a cigarette. The term secondhand smoke (SHS) illustrates this situation. According to research, SHS contains more than thousands of chemicals, of which hundreds are toxic and about seventy are carcinogenic, and has become an important environmental pollutant [11].

Studies have shown that smoking and SHS are both risk factors for KSD [12, 13]. But there is no comparison of the risks between smoking and SHS. The aim of present study was to examine the effects of smoking and SHS on the development of KSD.

## Methods

### Data Source, Study Population and Institutional Review Board Statement

This is a longitudinal cohort study, and all the participants were collected from Taiwan Biobank (TWB) which was designed for researchers to study the associations between environmental factors, clinical characteristics, personal health habits and disease. The detailed information for TWB has been described previously, and we will briefly explain it [14–16]. TWB is a community-based large-scale research database consisting of volunteers enrolled in 29 recruitment centers around Taiwan since 2008 with long-term follow-up. All volunteers in the present study underwent serial questionnaire surveys, physical examination and biospecimen collection every 2 years from 2008 until now and written informed consent was obtained in all subjects. The Declaration of Helsinki was followed by all investigations and this study was approved by our institute (KMUHIRB-E(I)-20,210,058). According to these principles, a total of 27,209 participants were enrolled in the present study at beginning, then those with known underlying KSD (N = 1,941) were excluded. Participants with missing age (N = 1), SHS data (N = 7), and smoking data (N = 4) were also excluded. The final analysis included 25,256 participants (**Supplementary Fig. 1**).

### Variables

We employed multiple sources to obtain the variables analyzed, including questionnaires, physical examinations, and blood tests. The questionnaires provided data on several factors, including age, gender, smoking and drinking habits, exercise routine, educational status, as well as medical history. Physical examinations were conducted to gather information on the body mass index (BMI), while the blood tests were conducted to obtain data on serum creatinine and uric acid levels, metabolic profiles (hemoglobin A1c, total cholesterol, triglyceride), and nutrient profiles (albumin, hemoglobin) that may affect the development of KSD [6], [15], [17–20]

### Smoking and SHS assessments

Information on smoking and SHS exposure was based on questionnaire surveys. Firstly, all participants were asked: “Have you ever smoked?” Participants that answered “No” were classified as “never-smokers, and those that answered “Yes” were classified as “ever-smokers”. Among never-smokers, participants were further asked “Have you been exposed to SHS for at least 5 minutes?” Never-smokers answered “Yes” were assigned as the ”never-smokers with SHS exposure group”, others were classified as the “never-smokers with no SHS exposure group.”

### Study outcome, incident KSD

The definition of KSD was based on self-reported diagnoses of KSD. Because we excluded all participants with a past history of KSD, the final study cohort had no KSD at baseline. During every follow-up, they were asked, “Have you ever had KSD?” The incident KSD was further defined as those that responded “Yes” to this question.

### Statistical analyses

We divided subjects into three groups: “never-smokers with no SHS exposure”, “ever-smokers”, and “never-smokers with SHS exposure” groups. Clinical characteristics of subjects in three groups including age, BMI, hemoglobin, albumin, hemoglobin A1c, total cholesterol, triglyceride, serum uric acid and serum creatinine were presented as mean ± standard deviation, while statistical significance of differences among these variables were assessed using one-way ANOVA. Other variables including gender, drinking, educational status, exercise, history of dyslipidemia, history of hypertension and history of diabetes were presented as numbers and percentages, and statistical significance of differences among these variables were assessed using Pearson χ [2] test. To determine the associations between smoking, SHS exposure and incident KSD, logistic regression analyses were used. Odd ratios (ORs) and 95% confidence intervals (CIs) were calculated before and after adjusting for confounders. The Box-Tidwell method was utilized to assess the linearity between the continuous variables and the logit of the dependent variable [21]. A Bonferroni correction was applied to all 27 model terms, with statistical significance accepted if the *p* value was less than 0.0018. [22] Following the evaluation, it was discovered that all continuous predictors had a linear connection with the logit of the dependent variable. Apart from verifying the linearity, three methods were used to assess the assumptions of the logistic regression model. The Hosmer-Lemeshow goodness-of-fit test, the omnibus test, and the residual analysis were used. A *p* value of less than 0.05 in the Hosmer-Lemeshow test indicates that the model lacks fit, whereas a *p* value greater than or equal to 0.05 suggests that the model fits well [23]. The omnibus test was utilized to determine the model’s overall significance in predicting the binary outcome, with a significant *p* value (less than 0.05) indicating the model’s predictive ability [24]. In the residual analysis, Cook’s D and leverage values were examined to identify any outliers in the model. Cook’s D [25] and leverage values [26] less than 1 suggest the absence of outliers in the regression model. R version 3.6.2 (R Foundation for Statistical Computing, Wien, Austria), Rstudio version 1.2.5033, and SPSS 20.0 (IBM Corp, Armonk, NY, USA) were the statistical tools and a *p* value of less than 0.05 was considered statistically significant.

## Results

### Baseline characteristics

There were 25,256 subjects in the longitudinal cohort. The mean age was 51 ± 10 years and 66% were women. A total of 17,872 (71%), 5,864 (23%), 1,520 (6%) subjects were included in the “never-smokers with no SHS exposure”, “ever-smokers”, and “never-smokers with SHS exposure” groups, respectively. The baseline characteristics of all subjects divided by presence of smoking and SHS exposure are summarized in Table 1. Compared with subjects in the “never-smokers with no SHS exposure” group, those in the “ever-smokers” group tended to be male gender, have higher prevalence of alcohol consumption, history of hypertension, history of diabetes mellitus, history of dyslipidemia and have higher BMI. Participants in the “never-smokers with SHS exposure” group tended to be younger, have higher prevalence of alcohol consumption and have higher BMI than those in the “never-smokers with no SHS exposure” group.

**Table 1:** Clinical characteristics of the study population (n = 25,256)

Characteristics	Never-smokers with no SHS exposure (N = 17,872)	Ever-smokers (N = 5,864)	Never-smokers with SHS exposure (N = 1,520)	*p* value
Age, yr	51 ± 10	51 ± 11	48 ± 10	< 0.001
Women, n (%)	14,452 (81)	1,157 (20)	1,159 (76)	< 0.001
Body mass index, kg/m^2^	23.7 ± 3.5	24.9 ± 3.5	24.2 ± 3.8	< 0.001
Alcohol status, ever, n (%)	457 (3)	1,497 (26)	113 (7)	< 0.001
Regular physical activity, n (%)	8,404 (47)	2,560 (44)	563 (37)	< 0.001
Educational status, n (%)				< 0.001
≦ High school	9,270 (52)	2,942 (50)	878 (58)	
≧Collage	8,602 (48)	2,922 (50)	641 (42)	
Hypertension, n (%)	2,008 (11)	905 (15)	162 (11)	< 0.001
Diabetes mellitus, n (%)	815 (5)	376 (6)	71 (5)	< 0.001
Dyslipidemia, n (%)	1,168 (7)	535 (9)	89 (6)	< 0.001
Hemoglobin, g/dl	13.4 ± 1.4	14.7 ± 1.4	13.4 ± 1.5	< 0.001
Albumin, g/dl	4.5 ± 0.2	4.6 ± 0.2	4.6 ± 0.2	< 0.001
Hemoglobin A1c, %	5.7 ± 0.7	5.8 ± 0.8	5.7 ± 0.9	< 0.001
Total cholesterol, mg/dl	196 ± 36	193 ± 35	194 ± 36	< 0.001
Triglyceride, mg/dl	106 ± 73	134 ± 101	110 ± 92	< 0.001
Uric acid, mg/dl	5.2 ± 1.3	6.2 ± 1.5	5.4 ± 1.4	< 0.001
Creatinine, mg/dl	0.7 ± 0.2	0.8 ± 0.4	0.7 ± 0.2	< 0.001
Follow-up, months	47 ± 14	47 ± 14	49 ± 15	< 0.001

SHS = secondhand smoke

### Association between smoking, SHS and the incident KSD

There were 642 (2.5%), 352 (2.0%), 240 (4.1%) and 50 (3.3%) participants had incident KSD during follow-up in all population, never-smokers with no SHS exposure, ever-smokers and never-smokers with SHS exposure respectively. As shown in Table 2, age, sex, BMI, alcohol consumption, hypertension, dyslipidemia, hemoglobin, albumin, hemoglobin A1c, triglyceride, uric acid and creatinine were significantly associated with KSD in univariable analysis (Table 2). The risk for KSD was also significantly higher in the ever-smokers (OR, 2.124; 95% CI, 1.798 to 2.510, *p* value < 0.001) and never-smokers with SHS exposure (OR, 1.693; 95% CI, 1.253 to 2.287, *p* value = 0.001) groups than in the never-smokers with no SHS exposure group. After adjusting for age, sex, BMI, smoking status, secondhand smoking, alcohol status, hypertension, dyslipidemia, hemoglobin, albumin, hemoglobin A1c, triglyceride, uric acid and serum creatinine, age, male gender, higher BMI, smoking and SHS exposure were five independent risk factors for the incident KSD (Table 3). Comparing the ever-smokers group with the never-smokers with no SHS exposure group, the OR for incident KSD showed significant increase in the ever-smokers group (OR, 1.282; 95% CI, 1.044 to 1.574, *p* value = 0.018). Comparing the never-smokers with SHS exposure group with the never-smokers with no SHS exposure group, the OR for incident KSD also showed significant increase in the never-smokers with SHS exposure group (OR, 1.662; 95% CI, 1.225 to 2.255, *p* value = 0.001). To further verify the assumptions of the logistic model, we conducted the Hosmer-Lemeshow goodness-of-fit test, the omnibus test, and residual analysis. These tests showed that the model fits well without outliers, as indicated in Supplementary Tables 1 and Supplementary Table 2.

**Table 2:** Odds ratios for incident KSD in univariate binary logistic analysis (n = 25,256)

Variables	Non-adjusted odds ratio (95% CI)	*P*
Age (per 1 year)	1.010 (1.003 to 1.018)	0.008
Female (vs. male)	0.394 (0.336 to 0.461)	< 0.001
Body mass index (per 1 kg/m^2^)	1.071 (1.050 to 1.092)	< 0.001
Alcohol status, ever (vs. never)	1.718 (1.360 to 2.171)	< 0.001
Regular physical activity, yes (vs. no)	1.006 (0.860 to 1.178)	0.937
Educational status, ≧Collage (vs. ≦High school)	1.051 (0.899 to 1.229)	0.534
Hypertension, yes (vs. no)	1.649(1.346 to 2.021)	< 0.001
Diabetes mellitus, yes (vs. no)	1.307(0.948 to 1.803)	0.103
Dyslipidemia, yes (vs. no)	1.440 (1.105 to 1.876)	0.007
Hemoglobin (per 1 g/dl)	1.298 (1.230 to 1.369)	< 0.001
Albumin (per 1 g/dl)	1.752(1.246 to 2.462)	0.001
Hemoglobin A1c (per 1%)	1.119(1.029 to 1.218)	0.009
Total cholesterol (per 1 mg/dl)	1.001(0.999 to 1.003)	0.419
Triglyceride (per 1 mg/dl)	1.002(1.001 to 1.002)	< 0.001
Uric acid (per 1 mg/dl)	1.265 (1.203 to 1.331)	< 0.001
Creatinine (per 1 mg/dl)	1.398 (1.199 to 1.628)	< 0.001
**Ever-smokers (**vs.**Never-smokers with no SHS exposure)**	**2.124 (1.798 to 2.510)**	**< 0.001**
**Never-smokers with SHS exposure (**vs.**Never-smokers with no SHS exposure)**	**1.693 (1.253 to 2.287)**	**0.001**

CI = Confidence interval; SHS = secondhand smoke

**Table 3:** Odds ratios for incident KSD in multivariate binary logistic analysis (n = 25,256)

Variables	Non-adjusted odds ratio (95% CI)	*P*
Age (per 1 year)	1.010 (1.001 to 1.018)	0.026
Female (vs. male)	0.569 (0.439 to 0.737)	< 0.001
Body mass index (per 1 kg/m^2^)	1.035 (1.010 to 1.061)	0.005
Alcohol status, ever (vs. never)	0.956 (0.742 to 1.231)	0.725
Regular physical activity, yes (vs. no)	-	-
Educational status, ≧Collage (vs. ≦High school)	-	-
Hypertension, yes (vs. no)	1.230(0.983 to1.538)	0.070
Diabetes mellitus, yes (vs. no)	-	-
Dyslipidemia, yes (vs. no)	1.089 (0.823 to 1.440)	0.551
Hemoglobin (per 1 g/dl)	1.065 (0.992 to 1.143)	0.081
Albumin (per 1 g/dl)	1.140(0.791 to 1.643)	0.482
Hemoglobin A1c (per 1%)	0.989(0.849 to 1.095)	0.833
Total cholesterol (per 1 mg/dl)	-	-
Triglyceride (per 1 mg/dl)	1.000(1.000 to 1.001)	0.229
Uric acid (per 1 mg/dl)	1.049 (0.981 to 1.122)	0.161
Creatinine (per 1 mg/dl)	0.948 (0.658 to 1.366)	0.776
**Ever-smokers (**vs.**Never-smokers with no SHS exposure)**	**1.282 (1.044 to 1.574)**	**0.018**
**Never-smokers with SHS exposure (**vs.**Never-smokers with no SHS exposure)**	**1.662 (1.225 to 2.255)**	**0.001**

CI = Confidence interval; SHS = secondhand smoke. Adjusted by age, gender, body mass index, smoking status, secondhand smoking, alcohol status, hypertension, dyslipidemia, hemoglobin, albumin, Hemoglobin A1c, triglyceride, uric acid and serum creatinine

### Odds ratios for incident KSD in multivariable binary logistic analysis in a subgroup of participants who had smoking or SHS exposure

To further examine the effects of smoking and SHS on KSD and to explore which has a greater impact on KSD, a subgroup analyses consisting participants who had smoking or SHS exposure were done. After adjusting for confounders, the odds to incident KSD were similar between never-smokers with SHS exposure and ever-smokers groups (Table 4). Compared the never-smokers with SHS exposure group with the ever-smokers, the OR for incident KSD did not showed significant increase in the never-smokers with SHS exposure group (OR, 1.223; 95% CI, 0.852 to 1.756, *p* value = 0.275) (Table 4).

**Table 4:** Odds ratios for incident KSD in multivariate binary logistic analysis in a subgroup of participants who had smoking or SHS exposure (n = 7384)

Variables	No. of incident KSD / No. of subjects (%)	Adjusted odds ratio (95% CI)	*P* value
Ever-smokers	240 / 5864 (4.1)	1.000 (Reference)	-
Never-smokers with SHS exposure	50 / 1520 (3.3)	1.223 (0.852 to 1.756)	0.275

CI = Confidence interval; SHS = secondhand smoke. Adjusted by age, gender, body mass index, smoking status, secondhand smoking, alcohol status, hypertension, dyslipidemia, hemoglobin, albumin, Hemoglobin A1c, triglyceride, uric acid and serum creatinine

## Discussion

We utilized a representative sample of the Taiwanese in a longitudinal study to examine the impacts of smoking and SHS on the development of KSD and found both factors were associated with a higher incidence of KSD. In addition, we were the first to explore that never-smokers with SHS exposure had similar effects on the development of KSD than smokers.

Smoking was reported to be associated with kidney stone formation in several studies. In a cross-sectional study of 232 patients, smoking was significantly associated with urinary stone (OR, 2.06; 95% CI, 1.06 to 4.01). [27] Another study analyzed 354 cases and 354 controls and concluded that smoking was an independent risk factor for KSD (OR, 1.66; 95% CI, 1.11 to 2.50). [28] Soueidan et al. also observed current smoker was strongly associated with risk of symptomatic urolithiasis (OR, 8.5; 95% CI, 2.2 to 32.2). [29] These articles are cross-sectional studies, while ours is longitudinal in that it provides additional evidence that smoking is associated with KSD.

There is only one article on the association between SHS and KSD. Chen et al. conducted a longitudinal study of the Taiwanese population and found that SHS was associated with a higher risk of KSD in a multivariable analysis adjusting for age, gender, lifestyle, comorbidities, and laboratory data (OR, 1.64; 95% CI, 1.21 to 2.23). [13] However, there was no research comparing the effects of smoking and SHS on the development of KSD. In the present study, the incidence of KSD was observed in 4.1% of ever-smokers and 3.3% of SHS exposures, with no significant difference between the groups. This shows that not only is smoking harmful to kidney health, but SHS as well. In this era of increasing emphasis on environmental safety, the establishment of a smoke-free environment is particularly important. Our findings support the argument by showing that the risk of KSD is similar between smokers and SHS exposures, and that policies against smoking and SHS should continue to be strengthened to promote global health.

The etiology of smoking and KSD is unclear. Some studies have hypothesized that smoking is associated with increased levels of vasopressin, which leads to decreased urine output, and contributes to stone formation [30–32]. Another proposed mechanism was the release of reactive oxygen species that may cause renal injury [33] Moreover, urinary calcium excretion, which is thought to be the protect factor of KSD, was lower in smoking patients [33]. It’s also found that increased levels of urinary metallic elements such as cadmium and mercury in smokers, which might be relevant to urinary tract stone formation [34, 35].

Apart from smoking and SHS exposure as risk factors for KSD in our study, age, male gender, and higher BMI are also independent risk factors. This result is compatible with the previous studies [4, 6, 36]. A cross-sectional study conducted in the southeast of Iran showed a high risk of kidney stone development for male gender (OR, 1.57; 95% CI, 1.39 to 1.76), higher BMI (25 to 29.9 kg/m^2^ OR, 1.24, 95% CI 1.11 to 1.40 and ≥ 30 kg/m^2^ OR: 1.30, 95% CI 1.14 to 1.48) [4]. Additionally, Taylor et al. found an association between obesity and KSD regarding gender, which shows the multivariable relative risk for stone formation in men with a BMI of 30 or greater compared with men with a BMI of 21 to 22.9 was 1.33 (95% CI 1.08–1.63), and the corresponding relative risks for the same categories of BMI in older and younger women were 1.90 (95% CI, 1.61–2.25) and 2.09 (95% CI, 1.77–2.48), respectively [36]. According to data from the nationwide, population based study in Taiwan, the age adjusted prevalence in 2010 in male and female subjects was 9.01% and 5.79%, respectively [37, 38]. The prevalence rate increased for each gender age group, with the peak prevalence in the 60 to 69-year age range (19.4%) [39]. These results are consistent with the findings of our study.

Our study has some limitations. Firstly, the KSD was diagnosed using a patient-based questionnaire, which may not be that precise. However, the patients’ self-reported KSD indicates that the disease has affected their lives, and that it is more representative of clinically significant KSD. Secondly, since there is no exposure time or duration of smoking and SHS, it is hard to quantify the exposure dosage of the relevant effects. Thirdly, no stone analysis results were collected in this study. Therefore, we were unable to explore the association of stone components with smoking and SHS. Fourthly, pregnant women and children are usually victims of SHS, but we didn’t explore the SHS effects on these individuals. This is an important topic that requires additional research to explore. Fifthly, due to the unavailability of incidence data on KSD in Taiwan, we were unable to apply the standard mortality ratio analysis in our study. Sixthly, we did not collect data on smokers who were also exposed to SHS, which was an oversight in the original study design. Lastly, smoking and SHS have long term effects on human health and long term follow up is needed.

## Conclusion

Our study suggests that both smoking and SHS are an independent risk factor for developing KSD and that the impact of SHS is not inferior to that of smoking, which raising the need for smoke-free environments.

## Electronic supplementary material

Below is the link to the electronic supplementary material.

Supplementary Material 1

## Data Availability

The data underlying this study are from the Taiwan Biobank. Due to restrictions placed on the data by the Personal Information Protection Act of Taiwan, the minimal data set cannot be made publicly available. Data may be available upon request to interested researchers. Please send data requests to Szu-Chia Chen, Division of Nephrology, Department of Internal Medicine, Kaohsiung Medical University Hospital, Kaohsiung Medical University.

## List of abbreviations

KSD: Kidney stone disease
SHS: secondhand smoke
TWB: Taiwan Biobank
ORs: Odd ratios
Cis: confidence intervals
BMI: Body Mass Index
