# Supplementary material for: The impact of secondhand smoke on the development of kidney stone disease is not inferior to that of smoking: a longitudinal cohort study
Source: BMC Public Health. 2023 Jun 20;23:1189. doi: 10.1186/s12889-023-16116-6 (PMC10283308; doi:10.1186/s12889-023-16116-6)
Supplement: Supplementary file 1 — Supplementary Material 1 [file 12889_2023_16116_MOESM1_ESM.docx]

**Supplementary Figure 1**. Study participants were classified by smoking and secondhand smoke exposure


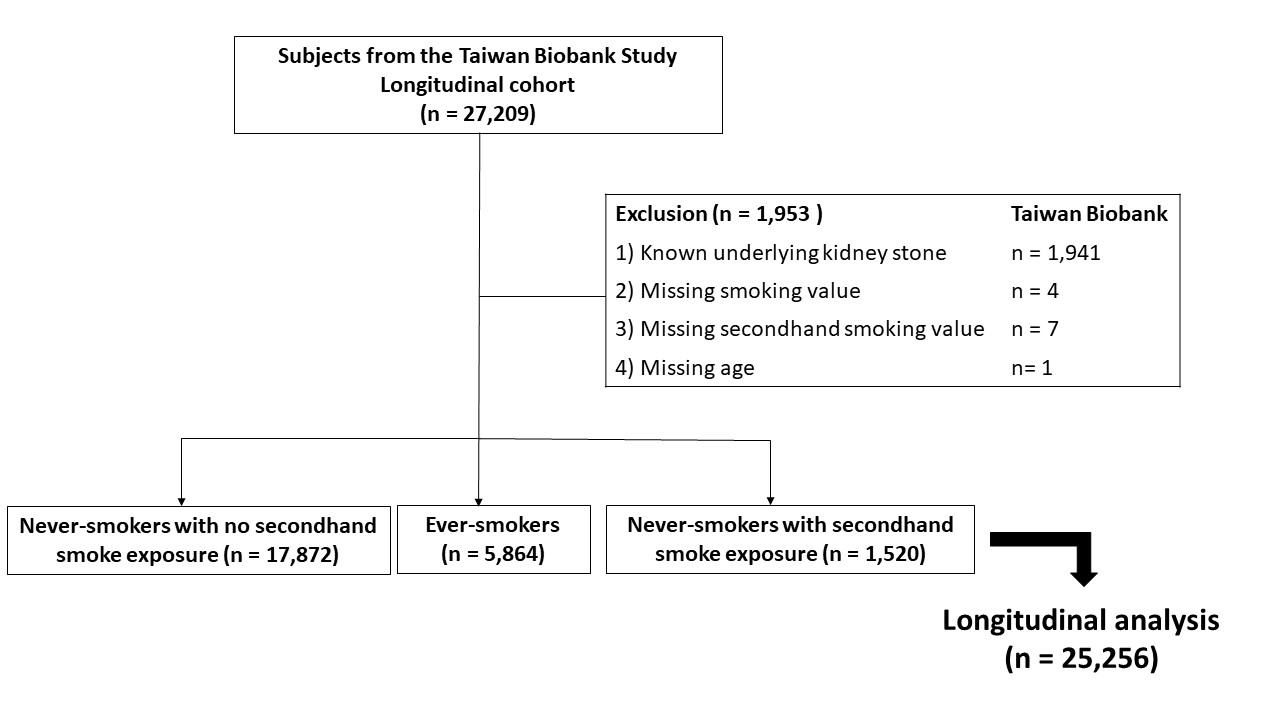


**Supplementary Table 1.** The Hosmer-Lemeshow goodness-of-fit test and the omnibus test for the prediction of the incident kidney stone disease.

|  | P value of the Hosmer-Lemeshow goodness-of-fit test | P value of the omnibus test |
| --- | --- | --- |
| Model | 0.925 | <0.001 |

* A p value less than 0.05 in the Hosmer-Lemeshow test indicates a lack of fit in the model, whereas a p value greater than or equal to 0.05 suggests that the model fits well. The omnibus test was used to determine whether the model as a whole was a significant predictor of the binary outcome, with a significant p value (less than 0.05) indicating the model's predictive ability.

**Supplementary Table 2.** The residual analysis for identifying any outliers in the model.

|  | Minimum | Maximum | Mean | Standard deviation |
| --- | --- | --- | --- | --- |
| Cook’s D value | 0 | 0.48097 | 0.00059 | 0.00537 |
| Leverage value | 0.0007 | 0.15842 | 0.00059 | 0.00175 |

* Cook's D and leverage values less than 1 indicate the absence of outliers in the regression model.
